# High risk of psychosis, condition or diagnosis? About a case

**DOI:** 10.1192/j.eurpsy.2025.2231

**Published:** 2025-08-26

**Authors:** M. L. Maria Del Carmen

**Affiliations:** 1 Psychiatric, Hospital General Universitario Gregorio Marañon, Madrid, Spain

## Abstract

**Introduction:**

A person with “high-risk mental status (HRMS)” indicates that the person, usually young people between the ages of 14 and 25, is more likely to develop psychosis. These people have attenuated psychotic symptoms without reaching the intensity or frequency of a frank psychotic episode. It is suggested that psychological trauma could favor neurochemical and psychopathological changes in a vulnerable individual. It would be interesting to study the role of psychotherapeutic interventions in the course of high-risk mental states and their possible evolution to a psychotic disorder.

We present the case of an 18-year-old adolescent whose diagnosis was high risk of psychosis.

**Objectives:**

This work has several objectives. On the one hand review current information on high-risk mental status (EMAR). On the other hand, develop a discussion about whether the EMAR category should be a diagnostic entity or just a condition.

**Methods:**

A bibliographic search has been carried out in the main sources of medical information such as Pubmed, Uptodate as well as in national and international journals. Likewise, the knowledge and clinical experience of the team has been reviewed in order to expose its own experience in this field, defining specific interventions as well as results.

**Results:**

The case presented is of an 18-year-old female patient. She states that the main reason for consultation is something that happened last Sunday, at which time he had “an identity crisis” in which he did not know if he was a girl or a boy. The reasoning behind this fact is that “as Pablo Alborán likes him, perhaps he is a boy”. Given the bizarreness of the explanation and the patient’s particular contact, I explore a previous psychopathological situation. She says that since last year she feels more insecure, with diffuse fear that it is difficult to specify or nominate something specific: “in class and that is very difficult for me, public presentations”, she says that “everything scares me”, she says that she has a non-specific fear that has been maintained even increasing over the months and that has led him to have greater anguish. Even though the patient dates the beginning of the picture on Sunday, it is noteworthy that the previous Thursday she had requested a consultation with psychology in the private circuit that although she does not know how to specify the reason “because of fears” it seems that the anguish resulting from this fear had been increasing, having greater difficulties for the presentations in class. The contact is psychotic and the situation that the patient describes is typical of a “treme” situation, cataloged in the current literature as a High-Risk Mental State.

**Conclusions:**

High-risk mental states are not a diagnostic category according to current classifications, although it is necessary to reach a consensus on what the diagnosis implies and what would be the way to proceed when a patient presents these symptoms.

**Disclosure of Interest:**

None Declared

